# Viscoelastic Study of Hemostasis Disorders Associated with *Echis ocellatus* Envenoming in North Benin Using a Quantra Analyzer

**DOI:** 10.3390/toxins17010003

**Published:** 2024-12-24

**Authors:** Sébastien Larréché, Roland Benes Chacha, Noé Sodjinou, Seidou Alassane Ouorou, Eric Ganhouingnon, Edith Aloukoutou Layo, Bruno Mégarbane, Achille Massougbodji, Jean-Philippe Chippaux

**Affiliations:** 1 Department of Medical Biology, Bégin National Military Teaching Hospital, F-94160 Saint-Mandé, France; 2 Inserm UMRS-1144, Université Paris Cité, F-75006 Paris, France; bruno.megarbane@aphp.fr; 3 Benin Clinical Research Institute, Abomey-Calavi, Benin; rolandchacha@gmail.com (R.B.C.); n.sodjinou9@gmail.com (N.S.); aloukoutoulayo@yahoo.fr (E.A.L.); massougbodjiachille@yahoo.fr (A.M.); 4 Emergency Department, Saint-Jean de Dieu Hospital, Tanguiéta, Benin; ouorouseidoua@gmail.com; 5 Medical Biology Laboratory, Saint-Jean de Dieu Hospital, Tanguiéta, Benin; ericganhouingnon@gmail.com; 6 Department of Medical and Toxicological Critical Care, Assistance Publique–Hôpitaux de Paris, Lariboisière Hospital, Federation of toxicology, F-75010 Paris, France; 7 French National Research Institute for Development, Mother and Child in Tropical Environment: Pathogens, Health System and Epidemiological Transition, Université Paris Cité, F-75006 Paris, France; jean-philippe.chippaux@ird.fr

**Keywords:** viscoelastic assay, Quantra, fibrinogen, coagulation, fibrinolysis, platelets, whole blood clotting test, antivenom

## Abstract

*Echis ocellatus* envenomings are a public health problem in West Africa, leading to bleeding and hypocoagulability. The aim of this study was to assess the hemostasis disorders associated with *E. ocellatus* envenoming. Envenomed patients with an abnormal whole blood clotting test (WBCT) were prospectively included at Tanguiéta, Benin. A WBCT with a sequential reading (i.e., at 20, 30, and 60 min), viscoelastic analysis (VA) using the Quantra analyzer, and blood count were performed on admission. VA and the WBCT were also assessed at 4, 8, 12, 24, 48, and 72 h after antivenom administration. Nineteen patients were included. On admission, the main results were an absence of a clot on VA and a slight decrease in platelets. Clot time gradually decreased over time while clot stiffness, fibrinogen, and platelet contributions to stiffness increased. Sequential reading improved the sensitivity of the WBCT. At H48, all patients with recurrence bleeding after antivenom administration had an abnormal WBCT while patients with a normal WBCT never had bleeding during their follow-up. VA allows the identification of various hemostasis disorders. Hypofibrinogenemia was the main disorder that persisted for several days after treatment. A WBCT with a sequential reading is an effective alternative for monitoring hypocoagulability in the absence of a laboratory.

## 1. Introduction

Snakebite is one of the most devastating neglected tropical diseases (NTDs) in sub-Saharan Africa, with 314,078 envenomings each year, of which 95% occur in rural areas, 7331 deaths, and between 5908 and 14,614 amputations [1], resulting in 1.03 million Disability-Adjusted Life Years (DALYs) [2]. In West Africa, the annual burden related to snakebite has been estimated at 4494 deaths, 5454 amputations, and 319,874 DALYs [3]. This makes it the fourth most important NTD in West Africa, after schistosomiasis, lymphatic filariasis, and rabies [3]. In Benin, household surveys have reported an average annual incidence of 440 bites per 100,000 inhabitants, with a mortality rate of 26 deaths per 100,000 inhabitants [4]. Snakebites occur mainly in two northern departments, Atacora and Borgou, during the rainy season [4,5].

The saw-scaled or carpet viper, *Echis ocellatus* Stemmler, 1970, is the main species involved in snakebite envenoming in West Africa, causing edema, spontaneous bleeding, blood hypocoagulability, necrosis, and acute kidney injury, leading to high mortality, of about 12–20% without antivenom therapy [3,6,7,8]. The hemorrhagic syndrome is characterized by bleeding at the bite site, then from mucous membranes (mainly gingival or genito-urinary localizations), recent or old scars, and even sometimes visceral or cerebral localizations [9,10,11,12]. Bleedings are due to the degradation of the basement membrane and the extracellular matrix surrounding the vessels by hemorrhagic snake venom metalloproteases (SVMPs) [13,14,15].

Hypocoagulability associated with *Echis* bites is the result of a venom-induced consumption coagulopathy (VICC) [16,17,18,19]. Whole *E. ocellatus* venom and some isolated SVMPs are capable of activating prothrombin to thrombin [18]. *Echis ocellatus* venom is also able to activate factor X in a calcium-dependent manner [20]. Fibrinogenases from *Echis* venom can directly cleave both the Aα- and Bβ- chains of fibrinogen [21].

In developing countries, the diagnosis of VICC is based on the use of the whole blood clotting test (WBCT), a simple, rapid, and inexpensive bedside test that can be used anywhere [9,22,23]. The principle is to detect the presence of a clot after collecting a few milliliters of venous blood from a bitten patient in a clean, dry, glass tube. The absence of a clot or the detection of an unstable clot indicates a coagulopathy. The test is typically read at 20 min [22,23,24], but other reading times ranging from 10 to 30 min have also been proposed [25,26]. An abnormal WBCT is associated with more severe envenoming, increased need for transfusion, and prolonged hospital stay [27].

VICC may also be confirmed by plasma-based coagulation assays such as increased prothrombin time (PT, sometimes expressed as International Normalized Ratio, INR), elevated activated partial thromboplastin time, or low fibrinogen [28,29]. Without effective treatment, these disorders can persist for a long time. In Djibouti, a spontaneous recovery of prothrombin time above 50% and fibrinogen level above 1 g/L occurs in 5.8 days and 7.5 days, respectively, after an *E. pyramidum* bite [29]. Increased D-dimers, fibrin(ogen) degradation products (FDPs), and soluble complexes, with decreased protein C and plasminogen, are consistent with hyperfibrinolysis [30,31,32,33]. Thrombocytopenia is less common than VICC in *Echis* envenoming [7,28,29]. The main limitation of plasma-based coagulation tests is their inconstant unavailability due to the lack of medical laboratories in the rural facilities where most patients are treated.

In contrast, viscoelastic tests can be used outside the laboratory [34]. Viscoelastic assays provide an overall assessment of clot formation and dissolution in real time because they are used with whole blood [35]. In the context of snakebite envenoming, these tests are capable of rapidly detecting the consumption of platelets, clotting factors, and fibrinogen, as well as platelet dysfunction or hyperfibrinolysis, and are more sensitive than a WBCT and plasma-based coagulation assays [36,37,38,39,40,41]. TEG has already demonstrated its ability to identify VICC in patients envenomed by *E. pyramidum* in Djibouti [36] while there is no similar experience in West Africa.

The Quantra hemostasis analyzer (HemoSonics, LLC, Charlottesville, VA, USA) is a new cartridge-based, automated system that uses ultrasound to assess the blood viscoelastic properties [42]. The technology is Sonic Estimation of Elasticity via Resonance (SEER) sonorheometry that can measure the dynamic evolution of a clot during the process of coagulation and fibrinolysis [43]. In a single test, the analyzer provides information on the clot coagulation time, clot stiffness, participation of fibrinogen and platelets in the clot, and fibrinolysis [44]. This analyzer has already been used in cardiac surgery [45,46,47], oncologic surgery [48], orthopedy [49], trauma [50], and COVID-19-associated coagulopathy [51], but never in snakebite envenoming.

The management of snakebite envenoming, and thus of the venom-induced hemorrhagic syndrome, is essentially based on antivenom [7,9,52,53]. Inoserp^TM^ PAN-AFRICA (Inosan Biopharma, Madrid, Spain) is a lyophilized polyvalent antivenom composed of highly purified F(ab’)_2_ immunoglobulin fragments obtained by the immunizing of horses with the venoms of *Echis ocellatus, E. pyramidum, E. leucogaster, Bitis gabonica, B. arietans, Naja haje, N. melanoleuca, N. nigricollis, N. pallida, Dendroaspis polylepis,* and *D. jamesoni* [52]. Each vial of antivenom neutralized at least 250 50% lethal doses of each venom. This antivenom was approved by the Benin Ministry of Health in 2013. Sequential administration of low doses of this antivenom, rigorously assessed every two hours for eventual renewal, is an efficient and well-tolerated strategy and can save antivenom [54,55]. Bleeding resolution was achieved in 60.7% of the patients within 2 h after the first injection of Inoserp^TM^ PAN-AFRICA in Cameroon. Resolution of a WBCT was achieved in less than 2 h for 75.2% of the patients and in less than 24 h in 96% of patients [54]. However, in some patients, persistence or recurrence of bleeding or even death due to bleeding has been reported despite antivenom treatment [52,54].

Studies carried out in patients envenomed by *E. pyramidum* in Djibouti showed a faster restoration of prothrombin time compared to that of fibrinogen, while hyperfibrinolysis could persist for several days [29,36]. On the other hand, there are few data on the evolution of hemostasis disorders after antivenom in patients envenomed by *E. ocellatus* in West Africa, mainly based on the WBCT [52]. Little is known about the recovery of platelets, clotting factors, fibrinogen, and fibrinolysis after antivenom treatment. In Nigeria, blood coagulability was restored between 2 and 39 h after antivenom. Fibrinogen reached 0.95 g/L at 24 h after treatment while hyperfibrinolysis suggested by an increase in fibrin degradation products was not corrected until one week [7]. A better understanding of the kinetics of these different hemostatic disorders would make it possible to optimize the treatment protocol and propose complementary therapies to the antivenom.

The aim of this prospective and observational study was to assess the following: (i) the hemostasis disorders in *E. ocellatus* snakebite patients presenting with bleeding or hypocoagulability on admission, using a WBCT with a sequential reading at 20, 30, and 60 min, viscoelastic test using Quantra analyzer, and blood count; and (ii) the changes in the WBCT and viscoelastic parameters after antivenom administration during the first three days.

## 2. Results

### 2.1. Description of Population on Admission

**Figure 1:**
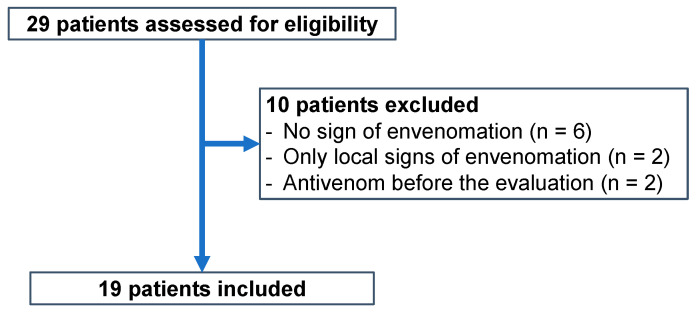
Flowchart for the inclusion in our study.

A total of 29 patients were screened for eligibility and 19 were included at the Saint-Jean de Dieu Hospital (HSJD), Tanguiéta, Benin (Figure 1).

Epidemiologic and clinical characteristics on admission are shown in Table 1 and Table 2. Patients had no significant medical history or comorbidity except for a previous snakebite reported by two patients. Three patients brought the snake that had bitten them to the hospital and all the snakes were identified as *E. ocellatus*. Systemic bleeding was present in 15 patients, while 4 patients had an abnormal WBCT without bleeding. No local necrosis or systemic neurotoxicity was reported.

### 2.2. Biological Parameters on Admission (H0)

For the whole blood clotting test (WBCT), seventeen patients had no clot regardless of the reading time. The test was discordant in two other patients: one patient had no clot at 20 min, then a normal clot at 30 and 60 min, while another patient had a normal clot at 20 min, a friable clot at 30 min, and an absence of a clot at 60 min. Thus, by pooling the results of the three reading times, all the patients had an abnormal WBCT. In comparison, all the patients with a dry bite had a normal clot at all three reading times.

A statistically significant decrease in platelets was observed in envenomed patients compared to the control group (*p* = 0.03) (Table 3). Thrombocytopenia was noted in six envenomed patients. Leukocytes and neutrophils were significantly increased in envenoming (*p* < 0.001 and *p* < 0.0001). The increase in neutrophils was significantly greater in patients with a dry bite than in the control group (*p* < 0.005).

In the Quantra analysis (Table 3), the clot time (CT) was significantly increased in envenomed patients (over 480 s), while the clot stiffness (CS), fibrinogen contribution to clot stiffness (FCS), and platelet contribution to clot stiffness (PCS) were collapsed and mostly unmeasurable by the analyzer. Because the CS was not measurable in most patients, the clot stability to lysis (CSL) could not be calculated.

### 2.3. Clinical Follow-Up and Treatment

**Figure 2:**
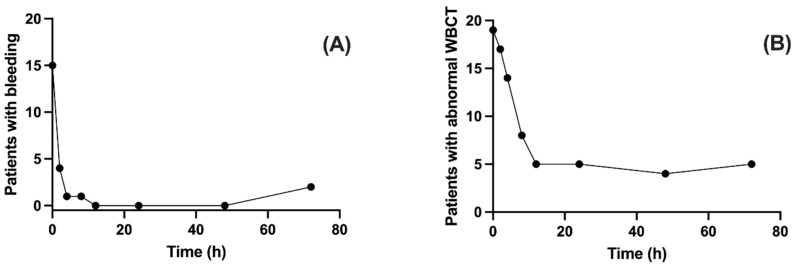
Proportion of patients with bleeding (**A**) and abnormal whole blood clotting time (WBCT) at 20, 30, and/or 60 min (**B**) on admission before antivenom (0), and after antivenom during the first three days in envenomed patients (n = 19).

All patients received a two-vials dose of antivenom on admission. Four patients still had mucosal bleeding at 2 h after the first administration (H2), with persistence also at H4 for one of the patients, then three other patients had recurrence of bleeding: one at H8 (subcutaneous hematoma) and two at H72 (mucosal bleeding) (Figure 2). A fourth patient had a recurrence of bleeding after the follow-up, on day 5 (subcutaneous hematoma).

Four patients received a new dose of antivenom: one at H2 then at H4, two at H72, and one after 72 h. Tolerance was good in the first hour, with only mild adverse events: one case of pruritus, two cases of nausea and vomiting, and one case of diarrhea, these symptoms not being described before antivenom administration. Subsequently, the adverse events were pruritus (two cases at H2, one case at H4, one case at H8), dizziness (one case at H4), and fever (one case at H24 and H48).

Several patients received transfusions: one patient received one unit of red blood cells at H4, then another at H24, and a final unit at H72; one patient received one unit of red blood cells at H48 and another at H72; and one patient received one unit of red blood cells after H72. One patient received fresh frozen plasma at H4 without a unit of red blood cells.

The length of hospital stay was 5 (IQ: 4–7) days and the longest stay was 13 days. There were no deaths.

### 2.4. Biological Follow-Up

From H12, most of the patients had a normal WBCT, but five patients still had an abnormal WBCT until at least H72 (Figure 2). On the day of discharge, all patients had a normal WBCT.

During the follow-up, 114 tests were performed on envenomed patients. There were 16 tests (14%) for which the result differed depending on the time of reading (Table 4). In 10 tests, the first reading at 20 min showed a normal clot. Of these 10 tests, the 30 min reading identified 6 abnormal WBCTs and the 60 min reading identified 4 additional abnormal WBCTs (for which the 20 min and 30 min readings found a normal WBCT). Five samples with a variable result between the different reading times found a friable clot at 20 min then no clot at 30 and/or 60 min. Finally, in one sample, the 20 min WBCT showed no clot, while the 30 min and 60 min WBCT showed a normal clot.

**Figure 3:**
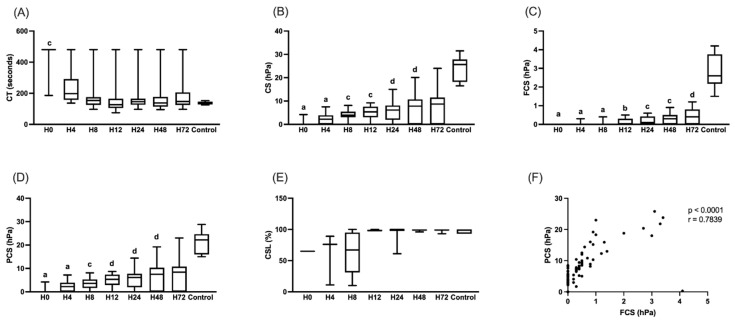
Evolution of clot time (CT, in seconds) (**A**), clot stiffness (CS, in hPa) (**B**), fibrinogen contribution to stiffness (FCS, in hPa) (**C**), platelet contribution to stiffness (PCS, in hPa) (**D**), and clot stability to lysis (CSL, in %) (**E**) on admission before antivenom (H0), at 4 (H4), 8 (H8), 12 (H12), 24 (H24), 48 (H48), and 72 (H72) hours after antivenom in envenomed patients (n = 19), and in healthy donors without a history of snakebite (control, n = 6). Results are expressed as medians and interquartile ranges. At H0, most points coincide with the median at 480 for CT and 0 for CS, FCS, and PCS. Parameters of envenomed patients were compared with those of the control group for each time point. a: *p* < 0.0001; b: *p* < 0.001; c: *p* < 0.01; d: *p* < 0.05. Spearman’s correlation between PCS and platelet count in envenomed patients at admission or during follow-up (n = 133) (**F**).

The CT gradually decreased over time, while the CS, FCS, and PCS increased (Figure 3). The CT was not significantly different compared to the control group from H4. In contrast, the CS was significantly lower than the control until H48, the FCS until H72, and the PCS until H48. The few values at H0, H4, and H8 for the CSL did not allow a statistical analysis but this parameter when it was measured was sometimes lower than that of the control group at H4 and H8. At H4, the three patients with a measurable CSL had a value less than 90%. At H8, four out of seven patients had a CSL less than 90%. Overall, five out of eight patients had a CSL compatible with hyperfibrinolysis. There was a strong correlation between the PCS and the FCS (*p* < 0.000; r = 0.784).

The three patients who had rebleeding after antivenom all had an abnormal global WBCT at H48 (Table 5). Previously, this test could have normalized or remained abnormal with antivenom. The patient who had rebleeding later on day 5 (number: 2,535,717) also had an abnormal global WBCT at H48. Thus, all patients with an abnormal global WBCT at H48 showed rebleeding. Conversely, all patients with a normal WBCT at H48 never had rebleeding during their follow-up or hospitalization. On Quantra analysis, the CT and CS were still not quantifiable at 48 h in three of the four patients who had rebleeding. In contrast, the CT was quantifiable in all patients without rebleeding, with a value less than or equal to 185 s. The CS was at 0 hPa at 48 h in two patients who never had rebleeding.

### 2.5. WBCT Versus Quantra Analyzer

**Figure 4:**
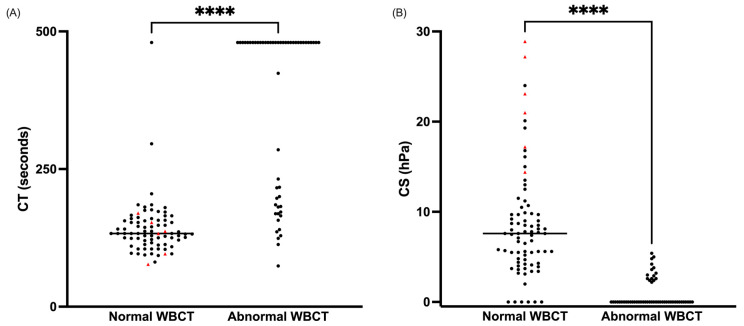
Clot time (CT in seconds) (**A**) and clot stiffness (CS in hPa) (**B**) of samples from envenomed (black circles) or dry bite (red triangles) patients (n = 133) based on the global whole blood clotting time (WBCT) result. The horizontal line corresponds to the median. Most points coincide with the median for CT and CS with abnormal WBCT. **** *p* < 0.0001.

An abnormal global WBCT was associated with a significantly increased CT and decreased CS (*p* < 0.0001) (Figure 4). The sensitivity of the global WBCT was 80% and 45.4% to detect a CT > 170 s and CS < 13 hPa, respectively. By pooling the three readings, the global WBCT had a better sensitivity to detect an increased CT than an isolated WBCT reading at 20, 30, or 60 min (Table 6). The sensitivity for detecting a decreased CS was also increased but remained low (Table 7).

## 3. Discussion

This study highlighted the multifocal action of the venom responsible for hypocoagulability during envenoming by *E. ocellatus*, with impairment of coagulation (particularly of fibrinogen), fibrinolysis, and platelets. To our knowledge, this is both the first study providing viscoelastic data on snakebites in West Africa and the first study using the Quantra analyzer in the context of snakebites. The advantage of sonorheometry over thromboelastography or rotational thromboelastometry is that it does not require direct contact with the sample. Therefore, there is no risk of interfering with clot formation in the early phase. This method is therefore theoretically more sensitive for detecting initial fragile clots [44].

Analysis with the Quantra analyzer revealed the same viscoelastic pattern as that described in patients envenomed by *E. pyramidum* in Djibouti, which is associated with a prolonged clotting time and decreased clot amplitude [36]. An increased CT reflects the consumption of clotting factors [47]. In patients envenomed by *E. ocellatus*, factors II, V, VII, VIII, X, XIII, and fibrinogen decrease [7,30,31]. A decreased CS depends on the number and function of platelets and the activity of fibrin formation [47,56]. Antivenom administration corrects the CT in the first 8 h while the CS remains significantly lower than the control for 48 h. The reduction in clot size seems to be mainly due to the decrease in the FCS. The correlation between the FCS and conventional Clauss fibrinogen level is strong [46]. Later correction of fibrinogen compared to other factors has already been reported in envenoming by *E. ocellatus* [7,57], *E. pyramidum* [29,36], and *E. carinatus* [32]. This difficulty in restoring fibrinogen could be due to liver damage *during E. ocellatus* envenoming. However, a clinical study of liver biomarkers found only a moderate increase in AST and GGT to twice the normal levels, while the ALT levels were comparable to those of the controls [58]. Administration of fresh frozen plasma may improve hypofibrinogenemia. Its association with antivenom has shown benefit in reducing the duration of coagulopathy in Australia [59] and India [60] but no similar study is currently available in sub-Saharan Africa.

Hyperfibrinolysis could also explain the slower correction of fibrinogen despite the antivenom. The CSL, which evaluates the level of fibrinolysis, was sometimes reduced only at H4 and H8. In most patients, this parameter was not measurable because the clot was too small. Therefore, it is impossible to know if this phenomenon is underestimated here. This result could be in favor of primary hyperfibrinolysis due to SVMP P-I with fibrino(geno)lytic activity [61,62] probably still present in the first hours after administration of the antivenom. On the other hand, the presence of soluble complexes and fibrin degradation products similar to those produced by plasmin in samples from patients envenomed by *E. ocellatus* in Nigeria suggests a possible secondary endogenous activation of the fibrinolytic system in response to venom-mediated consumptive coagulopathy [7,33]. During *E. pyramidum* envenoming in Djibouti, this enhanced fibrinolysis is sometimes described up to 72 h after the onset of antivenom administration [36]. Its effect on fibrinogen levels and bleeding remains unknown, while antifibrinolytics are not currently recommended for the treatment of snakebite hemorrhagic syndrome. Actually, no robust study has evaluated this treatment in this context, although its benefit has been widely demonstrated in other settings, such as the management of severe trauma-induced coagulopathy [63,64]. It could therefore be appropriate to evaluate the interest of antifibrinolytics in the management of snakebite-related bleeding.

The PCS could correspond to a quantitative and/or qualitative platelet function [46]. The PCS is very low during the first 48 h while moderate thrombocytopenia affects only a minority of patients. The rarity of thrombocytopenia has already been reported in other studies of *Echis* envenoming in Africa [7,29,36] although the activity of some hemorrhagic SVMPs isolated from *E. ocellatus* venom was able to activate washed human platelets in the absence of any additional cofactors [18]. In this case, the reduced PCS was more indicative of a qualitative impairment of platelets. Other SVMPs from *E. ocellatus* venom inhibit collagen-induced platelet aggregation [18]. Ocellatin [65] and ocellatusin [66], disintegrins isolated from *E. ocellatus* venom, interact with the glycoprotein IIb/IIIa complex, a fibrinogen-binding platelet receptor and inhibit ADP-induced platelet aggregation. Echicetin, a C-type lectin protein isolated from *E. carinatus* venom, is an agonist of platelet glycoprotein Ib, which is involved in von Willebrand factor binding and platelet adhesion, resulting in the inhibition of platelet plug formation [67]. Aggregometry or the measurement of a marker of platelet activity such as P-selectin should be used in order to confirm a potential thrombopathy. A strong correlation has been found between the PCS and the FCS. Thus, the decrease in the PCS may also be related to the consumption of fibrinogen, which is a major agonist of platelet aggregation [68].

The main limitation of viscoelastic tests is their cost, which, despite their clinical interest and their ease of use, would be a considerable obstacle to their deployment in peripheral health facilities to diagnose hemostasis disorders associated with snakebites.

Compared to modern hemostasis tests such as conventional plasma-based coagulation assays (prothrombin time or activated partial thromboplastin time) and viscoelastic tests (thromboelastography, rotational thromboelastometry, or SEER sonorheometry), the principle of the WBCT appears simple at first glance: a small amount of whole blood is collected from the bitten patient and then placed in a clean, dry, glass tube. After a certain period of time, the formation of a clot indicates the absence of snakebite-related coagulopathy [12,69]. This simple finding is actually the culmination of a complex phenomenon that combines the activation of various plasma proteins including fibrinogen, thrombin burst, platelet activation, and balanced fibrinolysis, the various stages of which are also assessed by viscoelastic tests [70]. If any of these steps are altered by snake venom, there is no clot or at least an abnormally friable clot. The only mechanism not investigated is the effect of SVMPs on the basement membrane of capillaries. Thus, although less sensitive than viscoelastic tests, the WBCT represents an interesting alternative because performed on whole blood, it provides an overall view of the patient’s hemostasis [40,41].

To improve the sensitivity of the test, we considered the WBCT as positive (i.e., abnormal) in the absence of a clot but also if the clot was friable or unstable, while the most common method, as described by the WHO, considers only the absence of a clot as a positive WBCT [24,71,72]. In addition, a sequential reading at different times (i.e., 20, 30, and 60 min) could provide dynamic information on global hemostatic function. The 30 and 60 min readings identified a hemostasis disorder in 11 samples in which the WBCT was normal at 20 min. Other samples with a variable result between the different time points showed a friable clot at 20 min and then a lack of a clot at 30 and/or 60 min. These two types of profiles were always associated with a very reduced CS (< 6 hPa) and can also reflect clot instability, although the CSL was mostly unavailable due to the small size of the clot. In contrast, the WBCT was abnormal at 20 min with an absence of a clot and then normalized from 30 min, probably reflecting a deficit in clotting factors. Thus, the global WBCT is more sensitive to detect a hemostasis disorder than a WBCT read at a single time, whatever it may be. Our results confirm the study by Benjamin et al., which showed the improvement in the diagnosis and monitoring of venom-induced coagulopathy with the inclusion of the friable clot observation and the sequential reading at 20 and 30 min [12]. In our study, the sensitivity of the global WBCT was 80% and 45.4% to detect a CT > 170 s and CS < 13 hPa, respectively. A meta-analysis reported an overall weighted sensitivity of 84% and 72% at detecting an INR > 1.4 and a fibrinogen level < 1 g/L, respectively [71]. In Nigeria, a prospective study in patients bitten by *E. ocellatus/romani* found a sensitivity of 87.2% for detecting an INR greater than or equal to 1.4 with a WBCT read at 20 min [24]. Our lower sensitivity than that reported in the literature can be explained by the fact that viscoelastometry itself is more sensitive than the INR, and by the fact that we chose a threshold value at the limit of the healthy population whereas an INR greater than 1.4 is obviously pathological. Other improvements to be considered to increase the sensitivity of the test would be to locally determine the coagulation time of the healthy population and to identify possible differences between children and adults or between women and men.

In the present study, a normal global WBCT better excluded clotting factor deficiency (prolonged CT) than fibrinogen deficiency (decreased CS and FCS), with an NPV of 86.1% and 17.7%, respectively. In the VICC described in envenoming by Australian elapids, effective clotting appears to occur with only minimal fibrinogen recovery to 0.5 to 1 g/L when the INR has nearly normalized [73]. The WBCT is therefore not very sensitive in detecting hypofibrinogenemia. Recent recommendations have suggested the PT/INR as the gold standard in the evaluation of snakebite-related coagulopathy [74]. Because of its good correlation with the CT, which reflects the PT/INR, a WBCT is therefore a good alternative for monitoring antivenom response in the absence of a laboratory. Furthermore, the decision to repeat a dose of antivenom is based on the persistence or recurrence of bleeding, and not on the existence of an isolated biological abnormality without bleeding.

No deaths or sequelae were noted in this study using the sequential antivenom administration proposed by Chippaux et al. [54]. In this procedure, additional doses of antivenom are administered only if bleeding (or neurological disorder in the case of elapid bite) persists or occurs. It conserves antivenom supplies by avoiding the unnecessary injection of vials. Therefore, all patients should be observed for several days in order to administer a new dose of antivenom if necessary, which is difficult in practice. Patients often want to leave the hospital as soon as possible to rejoin their families and return to work. An isolated prolonged WBCT is not considered as an indication to repeat the antivenom. However, an abnormal WBCT at 48 h would identify patients at high risk of relapse, which would encourage continued monitoring. On the other hand, patients with a normal 48 h test could be discharged from the hospital. This finding needs to be confirmed in a study with a larger number of patients.

This study has several limitations. The number of patients is quite modest due to its monocentric nature over a short period of time. Expanding future studies to include a larger, multicenter cohort would increase the reliability and power of the outcomes. On the other hand, the likely involvement of a single species makes the population fairly homogeneous. The protocol did not provide for analysis by Quantra beyond 72 h, whereas hemostasis disorders may persist beyond this period, given the results of a subsequent WBCT. The PT or INR was not included for in the protocol although it is now the reference criterion for the evaluation of snakebite-related coagulopathy [74]. Apart from the fact that this study was performed before the article by Abouyannis et al., the CT of the Quantra correlates relatively well with the PT.

## 4. Conclusions

The hypocoagulability associated with *E. ocellatus* envenoming is multifactorial, with the consumption of platelets, clotting factors and fibrinogen, platelet hypoaggregability, and hyperfibrinolysis. Hypofibrinogenemia appears to be a major determinant of the restoration of hemostasis under antivenom. The Quantra allows a comprehensive assessment of these hemostasis disorders and suggests ancillary treatment in conjunction with antivenom such as fresh frozen plasma or antifibrinolytics. A global WBCT with a sequential reading is an effective alternative for diagnosing and monitoring this hypocoagulability in the absence of a laboratory. WBCT-based monitoring could help identify patients without risk of rebleeding, allowing them to be discharged from the hospital sooner.

## 5. Materials and Methods

### 5.1. Ethical Statement

This study was approved by the National Ethics Committee for Health Research of Benin (approval number N°101/MS/DC/SGM/CNERS/SA of 13 June 2022). All participants and their parents/guardians (for minors) provided written and informed consent.

### 5.2. Data Collection

This study was carried out at the HSJD, Tanguiéta, Benin, between August and October 2022. Tanguiéta is located in Atacora, a mountainous region in the northwest of the country where there is a high incidence of bites by *E. ocellatus* [4,75].

Patients with a history of snakebite envenoming, age > 12 years, and no previous antivenom therapy who presented with bleeding and/or hypocoagulability defined by an abnormal WBCT were included in the study and treated according to the protocol (see below).

A standard form was completed with patient characteristics: gender, age, presence of comorbidities, geographic location of bite, bite site, time taken to reach the HSJD, snake identification, size of edema, necrosis, local or systemic bleeding, other clinical manifestations, laboratory tests, and treatments (number of antivenom vials, date and time of administration, transfusion). These data were then transferred to a secure website (www.easymedstat.com).

Clinical severity scores were used to measure the extent of edema (0 = no edema, 1 = edema confined to only one joint, 2 = edema extending to two joints, 3 = edema of all limbs except the root, 4 = edema extending beyond the root) and bleeding (0 = no bleeding, 1 = persistence of bleeding at the bite site for more than thirty minutes, 2 = bleeding of mucous membrane or recent scars, 3 = subcutaneous bleeding away from the bite site or old scars, 4 = externalization of internal bleeding or central nervous system bleeding). Hematuria and proteinuria were measured by dipstick.

The snakes responsible for the envenoming that were brought to the HSJD were identified by a trained herpetologist. In this region of Benin, the only species responsible for coagulopathy with an abnormal WBCT is *E. ocellatus*. Even if the snake was not brought, we considered it to be *E. ocellatus* if the patient had an abnormal WBCT on admission.

Inoserp^TM^ PAN-AFRICA (Inosan Biopharma, Madrid, Spain) was lyophilized and reconstituted in a 10 mL volume of an injectable saline solution. The protocol called for direct intravenous administration of (i) one vial for edema of any size, in the absence of hypocoagulability diagnosed by WBCT, bleeding, or neurological disorder; (ii) two vials for hypocoagulability and/or local bleeding lasting more than 30 min or systemic; (iii) four vials for neurological disorders. Clinical assessment was carried out one hour after administration to record the signs of intolerance. Any symptoms appearing after administration or re-administration of Inoserp^TM^ PAN-AFRICA and suggesting intolerance were considered as adverse effects.

A new clinical evaluation was performed at H2, H4, H8, H12, H24, H48, H72 after the first antivenom administration and on the day of discharge. At each evaluation, the evolution of the envenoming was measured according to the same clinical score as at the time of admission for bleeding.

A new dose of Inoserp^TM^ PAN-AFRICA was administered if bleeding persisted (two vials) or neurological disturbances occurred (four vials) at any assessment time.

### 5.3. Biological Analysis

Blood samples for WBCT, blood count, and viscoelastic analysis were collected on admission, after inclusion, and before antivenom. Five patients of both genders, presenting with a snakebite with no clinical signs or abnormal WBCT, constituted the “dry bite” group for biological analysis. In addition, 6 and 10 healthy donors of both genders, with no history of snakebite, constituted the “control” group for Quantra analyzer and blood count, respectively.

Patients with bleeding and/or hypocoagulability at H0 were also sampled at H2, H4, H8, H12, H24, H48, H72, and the day of discharge. At H2 and the day of discharge, a WBCT was performed. A WBCT and a viscoelastic analysis were carried out at the other sampling times.

A WBCT was determined as previously described [12,25,69]. Approximately two milliliters of venous blood was transferred directly into a clean and dry glass tube that was kept upright, open, and undisturbed at room temperature. The WBCT was assessed at three reading times: 20, 30, and 60 min. At each reading time, the quality of the clot was scored (0 = normal clot, 1 = fragmented or fragile clot (friable clot), 2 = absence of clot). WBCT was considered abnormal if the clot was scored 1 or 2.

Blood count was performed on 1% EDTA whole blood using a Yumizen H500 OT hematology analyzer (HORIBA medical, Montpellier, France). The parameters evaluated were leukocytes, neutrophils, hemoglobin, and platelets. Thrombocytopenia was defined by a platelet count below 150 G/L.

Viscoelastic analysis was performed with a Quantra hemostasis analyzer (HemoSonics, LLC, Charlottesville, VA, USA) that was loaned to the study. SEER sonorheometry uses high-frequency ultrasound pulses to create a shear wave that causes the blood sample to resonate. The vibration-induced oscillatory motion of the sample can be measured by the return echoes of a series of low-energy ultrasound tracking pulses. The shape and frequency of this vibration are directly related to the shear modulus of the sample. Repeated acquisitions every 4 s produce a time-displacement curve that shows the dynamic changes in the properties of the sample during coagulation [44]. The QStat cartridge (HemoSonics, LLC, Charlottesville, VA, USA) measures CT (in seconds) with intrinsic activation via kaolin; CS (in hPa) through extrinsic activation by tissue factor and polybrene; and FCS (in hPa) with tissue factor activation in addition to abciximab to block platelets and polybrene. Two additional parameters are reported as follows: PCS (in hPa) defined as the difference between the direct measures of CS and FCS, and CSL (in %), defined as the normalized difference between the clot stiffness change after maximum clot stiffness in the absence of tranexamic acid and the corresponding clot stiffness change in the presence of tranexamic acid, highlighting the influence of fibrinolysis [44]. If CT was > 480 s on the analyzer, CT was assigned a value of 480 s, CS, FCS, and PCS were assigned a value of zero, and no value was assigned to CSL. If CT was ≤ 480 s and CS not rendered by the analyzer, CS, FCS, and PCS were assigned a value of zero. If CSL was not rendered by the analyzer, no value was assigned to CSL.

### 5.4. Statistical Analysis

One investigator performed data entry using Easymedstat (version 3.27; www.easymedstat.com). Statistical analysis was performed using GraphPad PRISM 9.5.0 (GraphPad Prism Inc., La Jolla, CA, USA).

Discrete outcomes were expressed as absolute frequencies, and numerical variables were expressed as medians and interquartile range. Normality and heteroskedasticity of continuous data were assessed using Shapiro–Wilk and Levene’s tests, respectively. Due to the small size and non-Gaussian distribution of our sample, we used nonparametric tests to compare continuous variables. Continuous outcomes were compared using Mann–Whitney U test to compare medians between two groups of data. Kruskal–Wallis’ test followed by Dunn’s multiple comparisons were used to compare variables with three or more groups. Two-tailed tests were used. Statistical significance of results was considered when *p* < 0.05.

Spearman’s correlation coefficient was calculated to measure the degree of association between PCS and FCS. The correlation coefficient was considered significant when *p* < 0.05. The correlation index (*r*) was used to categorize the strength of the correlation as weak (*r* ≤ 0.35), moderate (*r* ≥ 0.36 to *r* ≤ 0.67), or strong (*r* ≥ 0.68), as previously described [76].

## Figures and Tables

**Table 1:** Epidemiological characteristics of envenomed patients. Numeric variables were expressed as median and interquartile range, and discrete outcomes as absolute frequencies.

Epidemiological Characteristics	Number of Patients
Gender: Male/female	12/7
Age in years: median (IQ)	24 (17, 30)
Time to snakebite admission in hours: median (IQ)	32 (7, 85)
Geographical location of bite	
Field	15
Route	2
Bush	1
Peridomicile or garden	1
Anatomical site of bite	
Foot	15
Hand	3
Leg	1

**Table 2:** Clinical features of envenomed patients on admission. WBCT: whole blood clotting test; * dipstick only performed in 17 patients. Discrete outcomes were expressed as absolute frequencies.

Clinical Features	Number of Patients
Edema	
Grade 1	3
Grade 2	11
Grade 3	5
Bleeding	
Grade 0	4
Grade 1	0
Grade 2	12
Grade 3	3
Hemorrhagic manifestations	
Gingival bleeding	8
Bleeding from recent scars	6
Persistent local bleeding	2
Subcutaneous bruising	2
Epistaxis	1
Sublingual hematoma	1
Submandibular hematoma	1
Urinary dipstick	
Proteinuria	14 *
Hematuria	8 *
Anormal WBCT	19
Other signs	
Pruritus	1
Nausea and vomiting	1
Hypotension	1
Dizziness	3
Malaise	6
Abnormal vision	1

**Table 3:** Biological parameters of envenomed patients and dry bite patients on admission before antivenom and healthy subjects (control group). CT: clot time in seconds; CS: clot stiffness in hPa; FCS: fibrinogen contribution to clot stiffness in hPa; PCS: platelet contribution to clot stiffness in hPa; CSL: clot stability to lysis in %; NA: not applicable. Due to the absence of a clot in envenomed patients, CSL was not measurable in these patients. Numeric variables were expressed as median and interquartile range for envenomed patients (*n* = 19), dry bite patients (*n* = 5) and control group (*n* = 10 for blood count and *n* = 6 for Quantra parameters). Mann–Whitney tests were used to compare envenomed and dry bite patients (^a^), envenomed patients and control group (^b^), dry bite patients and control group (^c^).

	Envenomed Patients	Dry Bite Patients	*p*-Value ^a^	Control Group	*p*-Value ^b^	*p*-Value ^c^
Blood count						
Platelets (G/L)	181 (133–215)	200 (140.8–272.8)	0.31	222 (191.8–267.5)	0.03	0.56
Leukocytes (G/L)	8.4 (6.5–11)	7.3 (5.4–9.5)	0.44	5.3 (4.5–6)	<0.001	0.12
Neutrophils (G/L)	5.1 (3.6–7.2)	3.4 (2.8–4.3)	0.09	2 (1.5–2.8)	<0.0001	<0.005
Hemoglobin (g/dL)	12.7 (10.9–14.7)	12.4 (10.8–13)	0.54	13.8 (12.4–14.9)	0.28	0.08
Quantra parameters						
CT (seconds)	480 (480–480)	135 (91.3–157.3)	<0.0001	138.5 (127.8–146)	<0.0001	0.94
CS (hPa)	0 (0–0)	22.1 (16.5–27.6)	<0.0001	25.6 (18.2–27.8)	<0.0001	0.7
FCS (hPa)	0 (0–0)	2.8 (1.4–3.2)	<0.0001	2.6 (2.2–3.7)	<0.0001	0.73
PCS (hPa)	0 (0–0)	19.2 (15.2–24.3)	<0.0001	22.2 (15-24-7)	<0.0001	0.7
CSL (%)	NA	97.5 (96.7–99.2)	NA	99 (93–100)	NA	0.78

**Table 4:** Whole blood clotting tests (WBCTs) for which the result was different depending on the reading time. WBCT 20: WBCT read at 20 min; WBCT 30: WBCT read at 30 min; WBCT 60: WBCT read at 60 min; CT: clot time in seconds; CS: clot stiffness in hPa; NP: not performed.

Sample	Visit Time	Bleeding	WBCT 20	WBCT 30	WBCT 60	CT (s)	CS (hPa)
1	H0	Yes	No clot	Normal clot	Normal clot	217	3.2
2	H0	Yes	Normal clot	Friable clot	No clot	185	4.2
3	H2	No	Normal clot	Normal clot	Friable clot	NP	NP
4	H2	No	Normal clot	Normal clot	Friable clot	NP	NP
5	H2	No	Friable clot	Friable clot	No clot	NP	NP
6	H4	No	Normal clot	Friable clot	Friable clot	157	0
7	H4	No	Friable clot	Friable clot	No clot	181	2.6
8	H4	No	Normal clot	Friable clot	Friable clot	200	3.8
9	H4	No	Friable clot	No clot	No clot	480	0
10	H4	No	Normal clot	Friable clot	No clot	136	0
11	H4	No	Friable clot	Friable clot	No clot	197	0
12	H8	No	No clot	Normal clot	Normal clot	480	3.6
13	H8	No	Normal clot	Normal clot	Friable clot	182	5.4
14	H8	No	Normal clot	Normal clot	Friable clot	129	4.8
15	H12	No	Normal clot	Friable clot	Friable clot	74	2.6
16	H24	No	Friable clot	Friable clot	No clot	172	2.6
17	H48	No	Normal clot	Friable clot	Friable clot	113	2.9
18	H72	Yes	Normal clot	Friable clot	Friable clot	165	0

**Table 5:** Course of global whole blood clotting test (WBCT, read at 20, 30, and 60 min) and parameters of Quantra analyzer (CT, clot time in seconds, and CS, clot stiffness in hPa) in patients with recurrent bleeding. Follow-up on admission before antivenom (H0), at 4 (H4), 8 (H8), 12 (H12), 24 (H24), 48 (H48), and 72 (H72) hours after antivenom. NP: not performed.

Patient	Parameter	H0	H4	H8	H12	H24	H48	H72
2329424	Systemic bleeding	No	No	Yes	No	No	No	No
	WBCT	Abnormal	Abnormal	NP	Abnormal	Abnormal	Abnormal	Abnormal
	CT (seconds)	>480	>480	NP	>480	>480	>480	>480
	CS (hPa)	0	0	NP	0	0	0	0
2750992	Systemic bleeding	Yes	No	No	No	No	No	Yes
	WBCT	Abnormal	Abnormal	Abnormal	Normal	Abnormal	Abnormal	Abnormal
	CT (seconds)	>480	200	182	113	172	>480	>480
	CS (hPa)	0	3.8	5.4	8.1	2.6	0	0
2762854	Systemic bleeding	Yes	No	No	No	No	No	Yes
	WBCT	Abnormal	Normal	Normal	Normal	Normal	Abnormal	Abnormal
	CT (seconds)	>480	148	113	132	125	113	165
	CS (hPa)	0	7.5	7.8	9.2	3.4	2.9	0
2535717	Systemic bleeding	Yes	No	No	No	No	No	No
	WBCT	Abnormal	Abnormal	Abnormal	Abnormal	Abnormal	Abnormal	Abnormal
	CT (seconds)	>480	285	169	>480	424	>480	>480
	CS (hPa)	0	0	0	0	0	0	0

**Table 6:** Comparison of sensitivity, positive predictive value (PPV), specificity, and negative predictive value (NPV) of the whole blood clotting time read at 20 min (WBCT20), 30 min (WBCT 30), 60 min (WBCT 60), and pooling the results of the three readings (global WBCT) for detecting a clot time > 170 s on Quantra analyzer (n = 133). Values are expressed as n/N (%).

	WBCT 20	WBCT 30	WBCT 60	Global WBCT
Sensitivity	41/55 (74.5%)	41/55 (74.5%)	42/55 (76.4%)	44/55 (80.0%)
PPV	41/45 (91.1%)	41/50 (82%)	42/52 (80.8%)	44/54 (81.5%)
Specificity	74/78 (94.9%)	69/78 (88.5%)	68/78 (87.2%)	68/78 (87.2%)
NPV	74/88 (84.1%)	69/83 (83.1%)	68/81 (84.0%)	68/79 (86.1%)

**Table 7:** Comparison of sensitivity, positive predictive value (PPV), specificity, and negative predictive value (NPV) of the whole blood clotting time read at 20 min (WBCT20), 30 min (WBCT 30), 60 min (WBCT 60), and pooling the results of the three readings (global WBCT) in ascertaining a clot stiffness < 13 hPa on Quantra analyzer (n = 133). Values are expressed as n/N (%).

	WBCT 20	WBCT 30	WBCT 60	Global WBCT
Sensitivity	45/119 (37.8%)	50/119 (42.0%)	52/119 (43.7%)	54/119 (45.4%)
PPV	45/45 (100%)	50/50 (100%)	52/52 (100%)	54 /54 (100%)
Specificity	14/14 (100%)	14/14 (100%)	14/14 (100%)	14/14 (100%)
NPV	14/88 (15.9%)	14/83 (16.9%)	14/81 (17.3%)	14/79 (17.7%)

## Data Availability

The original contributions presented in this study are included in this article. Further inquiries can be directed to the corresponding authors.
